# Pulsed addition of HMF and furfural to batch-grown xylose-utilizing *Saccharomyces cerevisiae* results in different physiological responses in glucose and xylose consumption phase

**DOI:** 10.1186/1754-6834-6-181

**Published:** 2013-12-16

**Authors:** Magnus Ask, Maurizio Bettiga, Varuni Raju Duraiswamy, Lisbeth Olsson

**Affiliations:** 1 Department of Chemical and Biological Engineering, Industrial Biotechnology, Chalmers University of Technology, SE-41296, Gothenburg, Sweden

**Keywords:** Lignocellulosic ethanol, HMF, Furfural, Redox metabolism, Energy metabolism, Transcriptome, *Saccharomyces cerevisiae*

## Abstract

**Background:**

Pretreatment of lignocellulosic biomass generates a number of undesired degradation products that can inhibit microbial metabolism. Two of these compounds, the furan aldehydes 5-hydroxymethylfurfural (HMF) and 2-furaldehyde (furfural), have been shown to be an impediment for viable ethanol production. In the present study, HMF and furfural were pulse-added during either the glucose or the xylose consumption phase in order to dissect the effects of these inhibitors on energy state, redox metabolism, and gene expression of xylose-consuming *Saccharomyces cerevisiae*.

**Results:**

Pulsed addition of 3.9 g L^-1^ HMF and 1.2 g L^-1^ furfural during either the glucose or the xylose consumption phase resulted in distinct physiological responses. Addition of furan aldehydes in the glucose consumption phase was followed by a decrease in the specific growth rate and the glycerol yield, whereas the acetate yield increased 7.3-fold, suggesting that NAD(P)H for furan aldehyde conversion was generated by acetate synthesis. No change in the intracellular levels of NAD(P)H was observed 1 hour after pulsing, whereas the intracellular concentration of ATP increased by 58%. An investigation of the response at transcriptional level revealed changes known to be correlated with perturbations in the specific growth rate, such as protein and nucleotide biosynthesis. Addition of furan aldehydes during the xylose consumption phase brought about an increase in the glycerol and acetate yields, whereas the xylitol yield was severely reduced. The intracellular concentrations of NADH and NADPH decreased by 58 and 85%, respectively, hence suggesting that HMF and furfural drained the cells of reducing power. The intracellular concentration of ATP was reduced by 42% 1 hour after pulsing of inhibitors, suggesting that energy-requiring repair or maintenance processes were activated. Transcriptome profiling showed that NADPH-requiring processes such as amino acid biosynthesis and sulfate and nitrogen assimilation were induced 1 hour after pulsing.

**Conclusions:**

The redox and energy metabolism were found to be more severely affected after pulsing of furan aldehydes during the xylose consumption phase than during glucose consumption. Conceivably, this discrepancy resulted from the low xylose utilization rate, hence suggesting that xylose metabolism is a feasible target for metabolic engineering of more robust xylose-utilizing yeast strains.

## Background

Lignocellulosic biomass is an abundant and renewable resource for sustainable bioethanol production that usually does not compete with food crops [1,2]. Although researchers have spent a lot of effort to make the production process industrially viable, there are several problems that remain to be solved. Two of the most important challenges involve broadening of the substrate range and improving the robustness of the cell factory [3,4]. The former originates from the fact that lignocellulose is a complex heteropolymer consisting of both pentoses and hexoses intertwined in a matrix of lignin, and to make the production process economically feasible, all sugars have to be converted to ethanol [5]. *Saccharomyces cerevisiae*, the commonly used cell factory for ethanol production in industry, lacks the endogenous ability to metabolize pentoses such as xylose and arabinose. Several metabolic engineering strategies have thus been devised for introducing xylose utilization pathways from other microorganisms into *S. cerevisiae*[6].

The demand for robust microorganisms for bioethanol production stems from the fact that lignocellulosic raw materials have to be pretreated prior to fermentation. The pretreatment process induces structural changes in the raw material that decreases recalcitrance and facilitates subsequent enzymatic hydrolysis. But since the pretreatment has to be carried out at harsh conditions (most methods use high temperatures and low pH), degradation products are formed or released that can inhibit microbial metabolism [7]. Apart from organic acids and phenolic compounds, the furan aldehydes 5-hydroxymethylfurfural (HMF) and 2-furaldehyde (furfural) have been identified as particularly challenging for microorganisms [8]. They are formed in dehydration reactions of hexoses and pentoses, respectively [9,10]. The physiological effects of HMF and furfural on several microorganisms have been studied extensively over the years (as reviewed by Almeida *et al.*[8]), but most physiological studies have been performed with glucose as the sole carbon source. Since less is known about how the inhibitory effects are exerted when xylose is used as substrate, the aim of the present study was to investigate the metabolic effects of furan aldehydes on xylose-consuming *S. cerevisiae* during xylose consumption. Studying physiological responses of yeast to HMF and furfural can provide hints on approaches to overcome inhibition through either enhancing or attenuating specific metabolic responses of the cells.

Two principal strategies have been exploited for the engineering of xylose-utilization in *S. cerevisiae*[11,12]*.* The first strategy, which was engineered in the strain investigated in the present study, makes use of the *Scheffersomyces stipitis* genes *XYL1* and *XYL2* coding for xylose reductase (XR) and xylulose dehydrogenase (XDH), respectively. In this pathway, xylose is first reduced to xylitol by XR using preferably NADPH for reducing power. Xylitol is then oxidized to xylulose by the NAD^+^-dependent XDH. The different cofactor preferences of XR and XDH result in xylitol accumulation due to the inability of the cells to reoxidize the formed NADH [11]. The second strategy involves xylose isomerase (XI), which converts xylose to xylulose directly, thus avoiding the redox-related accumulation of xylitol.

Xylose utilization has been demonstrated to provide less energy in the form of ATP compared to when glucose is used as a substrate [13]. As stress responses to endogenous toxic metabolites require energy for maintenance, it can be hypothesized that inhibitors such as HMF and furfural can have a more pronounced effect when xylose is used as substrate. In fact, it has been demonstrated in studies with the lignocellulose-derived inhibitor acetic acid that the toxic effects are severely enhanced when xylose serves as the sole carbon and energy source rather than glucose. The inhibitory effect of acetic acid originates from the increased influx of protons over the cell membrane, which has to be counteracted by an ATP-dependent efflux to maintain the intracellular pH at a constant level [14]. The increased toxicity of acetic acid during growth on xylose was attributed to the low ATP production rate during xylose consumption and could be alleviated by a limiting glucose feed [13]. Correspondingly, we were interested in how HMF and furfural would affect energy metabolism when xylose is used as the sole carbon and energy source.

Under anaerobic conditions, *S. cerevisiae* can reduce HMF and furfural to their less toxic corresponding alcohols, HMF alcohol and furfuryl alcohol [15,16]. This conversion is advantageous for the cells, since furan aldehydes have been shown to inhibit several enzymes in glycolysis [17], decrease the specific growth rate [18], and induce reactive oxygen species (ROS) [19] in yeast. The conversion of furan aldehydes to alcohols is performed by oxidoreductases using NAD(P)H for reducing power. As NAD(P)H is used in a plethora of intracellular redox reactions, perturbations in the cofactor levels can result in cell-wide effects [20]. In fact, we recently demonstrated that the [NADH]/[NAD^+^] and [NADPH]/[NADP^+^] ratios were significantly decreased in chemostat cultivations with xylose-utilizing *S. cerevisiae* where HMF and furfural were added to the feed medium, compared to controls with no addition of inhibitors, indicating that the furan aldehydes were draining the cells of reducing power as a long-term stress reaction [21]. In the present study, we focused on how HMF and furfural affect redox metabolism when xylose was present as the sole carbon source, as compared to when glucose was available as the carbon and energy source.

To investigate the effects of HMF and furfural on energy and redox metabolism, we measured the intracellular concentrations of the adenonucleotides ATP, ADP, and AMP and the redox cofactors NAD(H) and NADP(H) of a xylose-utilizing *S. cerevisiae* strain in anaerobic batch cultivations on glucose-xylose mixtures after pulsed addition of furan aldehydes in either the glucose or the xylose consumption phase. To investigate short-term effects in gene expression following inhibitor pulses in the glucose and xylose consumption phases, transcriptome analyses were performed. To the authors’ best knowledge, the present study is the first thorough investigation of the impacts of HMF and furfural on energy state, redox metabolism, and gene expression during xylose conversion in a short-term perspective.

## Results

To investigate the transient metabolic responses caused by the presence of furan aldehydes, HMF and furfural were pulse-added to batch cultivations of xylose-consuming *S. cerevisiae*. As the carbon source, and thereby the energy generation, may influence the ability of the cells to cope with stress, inhibitors were pulsed in the glucose and xylose consumption phases, respectively. Changes in metabolic yields, redox and energy state, and transcriptome were then quantified. As glucose is the preferred substrate for *S. cerevisiae*, consumption of glucose and xylose occurs sequentially. Hence, in the following sections, the metabolic effects of pulsing HMF and furfural in the glucose consumption phase will be presented first, followed by the effects after pulsing in the xylose consumption phase. Finally, a comparison of the responses evoked by furan aldehydes in the two phases will be described.

### Inhibitor pulse in glucose consumption phase

#### Changes in local yields of metabolic products

To provide a benchmark for inhibitor responses in the xylose consumption phase, HMF and furfural at 3.9 and 1.2 g L^-1^, respectively, were pulsed into anaerobic batch cultivations in the exponential growth phase on glucose when approximately half of the glucose present initially had been consumed. The ratio between HMF and furfural were based on concentrations that are common in spruce hydrolysates [22], and the absolute concentrations were non-lethal, but significantly influencing the cells, as determined from preliminary experiments (data not shown).

**Figure 1:**
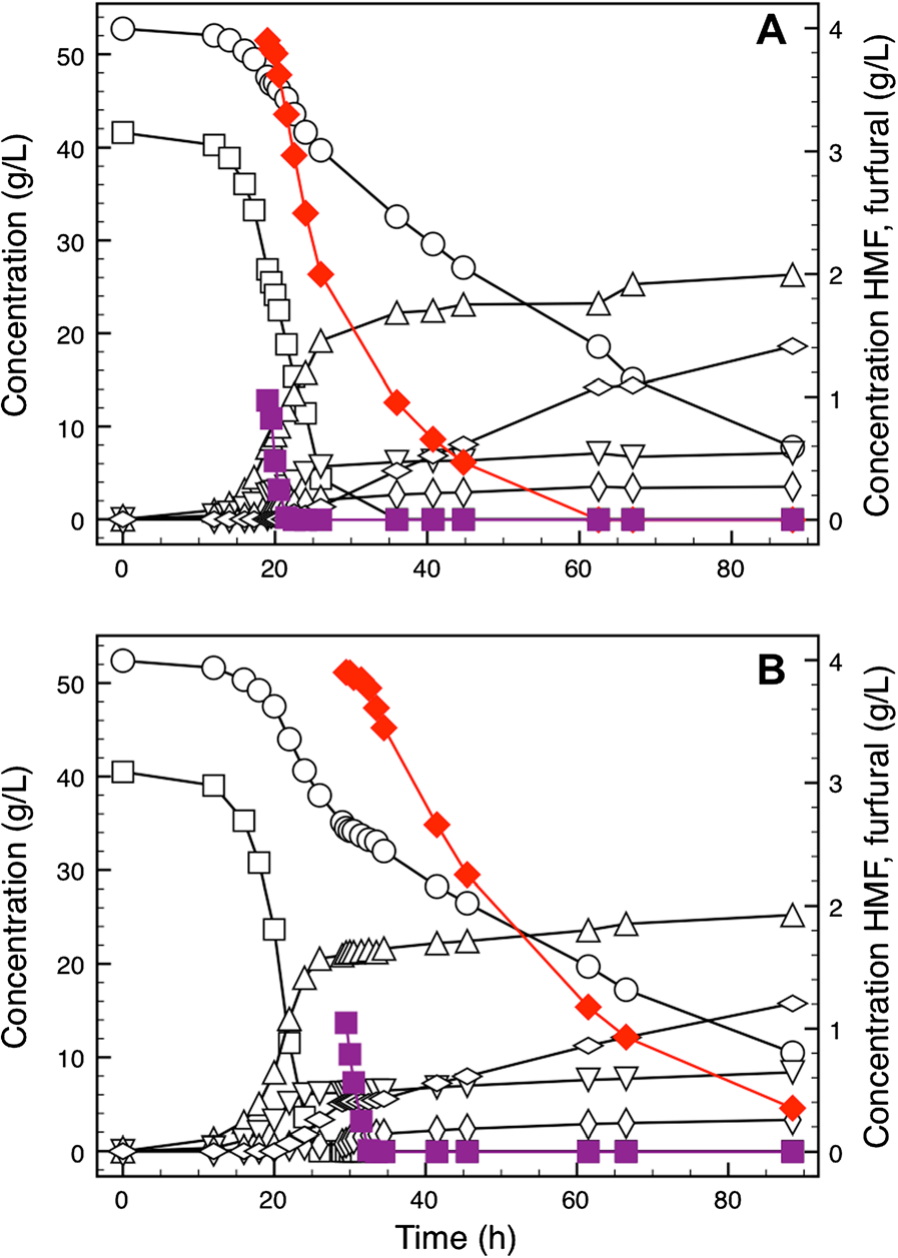
**Time course of sugar, metabolite, and inhibitor concentrations after pulsing of inhibitors in the glucose and xylose consumption phase.** After the inhibitor pulse, the response was divided into three phases in the two experiments. **(A)** For the cultivations pulsed during glucose consumption, the phases were defined as: glucose phase (12 to 17.25 hours), co-conversion phase (19 to 22.5 hours), and HMF phase (22.5 to 26 hours). **(B)** For the cultivations pulsed during xylose consumption, the phases were defined as: xylose phase (26 to 29.5 hours), co-conversion phase (29.5 to 32.5 hours), and HMF phase (33.5 to 88.5 hours). Squares, glucose; circles, xylose; triangles, ethanol; inverted triangles, glycerol; elongated diamonds, acetate; compressed diamonds, xylitol; red diamonds, HMF; purple squares, furfural. HMF: 5-hydroxymethylfurfural.

**Figure 2:**
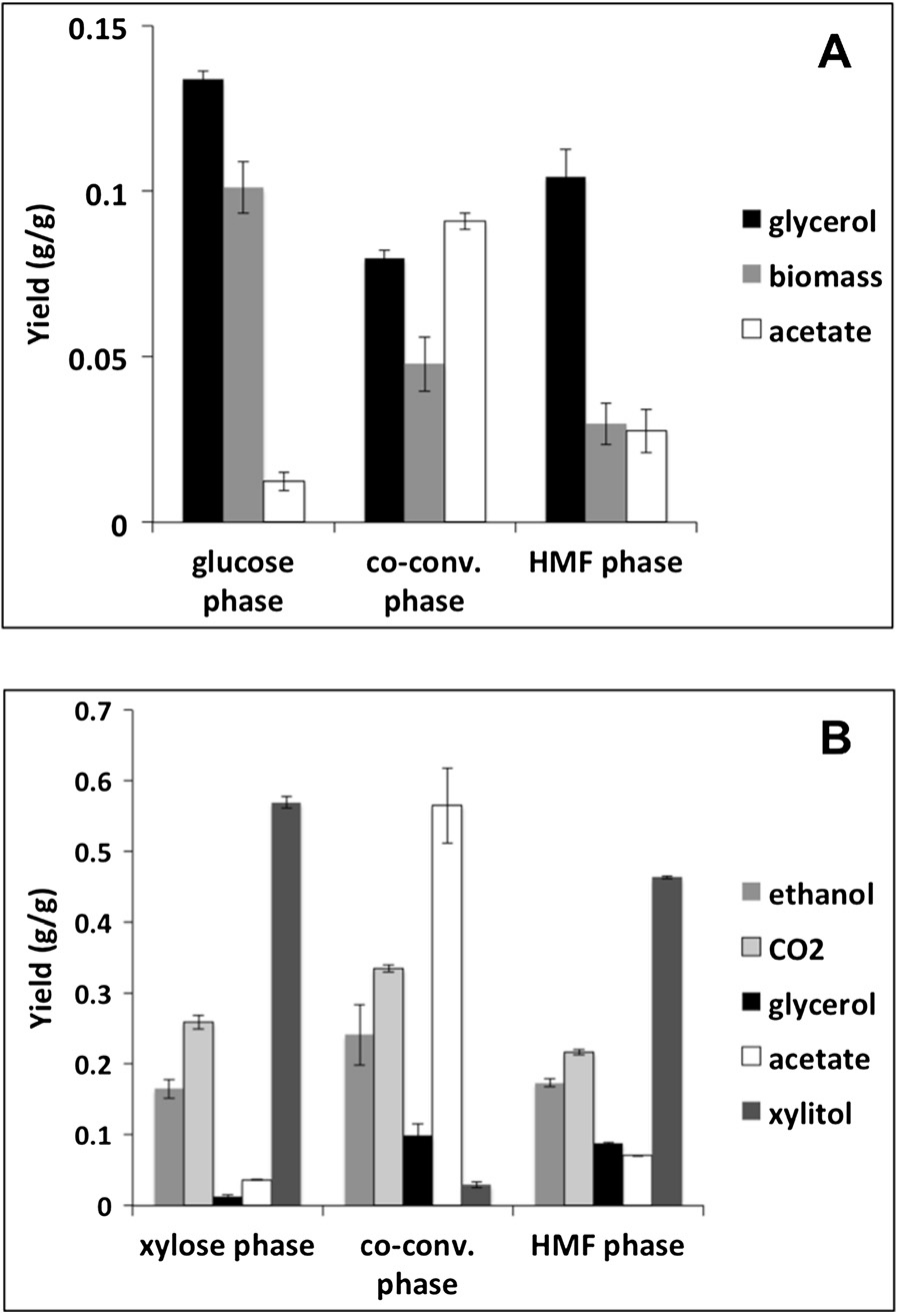
**Local yields calculated following inhibitor pulse in the glucose consumption phase and xylose consumption phase. (A)** Glucose consumption phase and **(B)** xylose consumption phase. The columns represent data calculated from three independent cultivations and the error bars indicate the standard deviations. HMF: 5-hydroxymethylfurfural.

The specific growth rate decreased from 0.32 to 0.09 h^-1^ immediately after addition of the inhibitors. Furfural was converted in 3.5 hours, whereas HMF had been completely converted 43.5 hours after pulsing (Figure 1A). The specific conversion rate of furfural was 2.6-fold higher than the specific conversion rate of HMF. To evaluate the transient metabolic response of the cells after pulsing, three different phases were defined: glucose phase (period before the pulse), co-conversion phase (period in which HMF and furfural were co-converted), and HMF phase (period when only HMF was converted and glucose was still available). As the fluxes of major metabolic products reflect the physiological responses, the local yields of biomass and primary metabolites were calculated in the different phases (Figure 2A). Interestingly, the glycerol yield decreased by 40% during the co-conversion phase. As furfural had been consumed, the glycerol yield increased, but was still 22% lower than in the glucose phase. The biomass yield decreased by 53 and 70% in the co-conversion and the HMF phases, respectively, compared to the glucose phase. Remarkably, the acetate yield increased 7.3-fold in the co-conversion phase, but decreased again during the HMF phase, though it was still 2.2 times higher than in the glucose phase.

#### Redox and energy changes following inhibitor pulse

**Figure 3:**
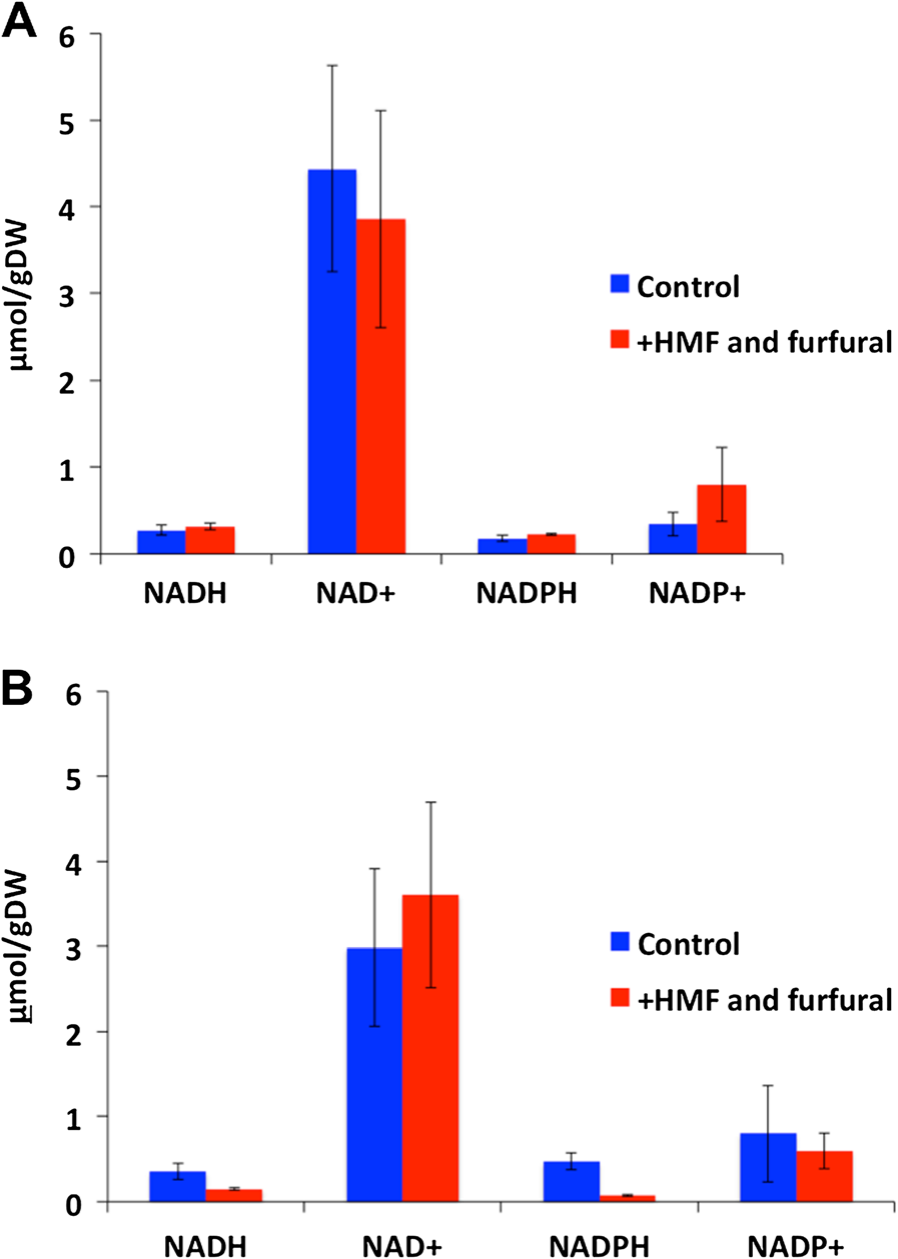
**Intracellular concentrations of the redox cofactors NAD(P)**^**+ **^**and NAD(P)H.** Intracellular concentrations of the redox cofactors NAD(P)^+^ and NAD(P)H 1 hour after pulsing of inhibitors in **(A)** the glucose consumption phase and **(B)** xylose consumption phase, respectively, compared with non-pulsed control cultivations. The columns represent data obtained from three independent cultivations and the error bars indicate the standard deviations. HMF: 5-hydroxymethylfurfural.

As the conversion of HMF and furfural under anaerobic conditions has been demonstrated to involve reductive reactions at the expense of NAD(P)H, the intracellular concentrations of the reduced and oxidized cofactors were determined 1 hour after the inhibitor pulse. The concentrations of the individual cofactors did not differ significantly between the pulsed and non-pulsed cultivations (Figure 3A). Consequently, the catabolic and anabolic reduction charges were similar for pulsed and non-pulsed cultivations.

**Figure 4:**
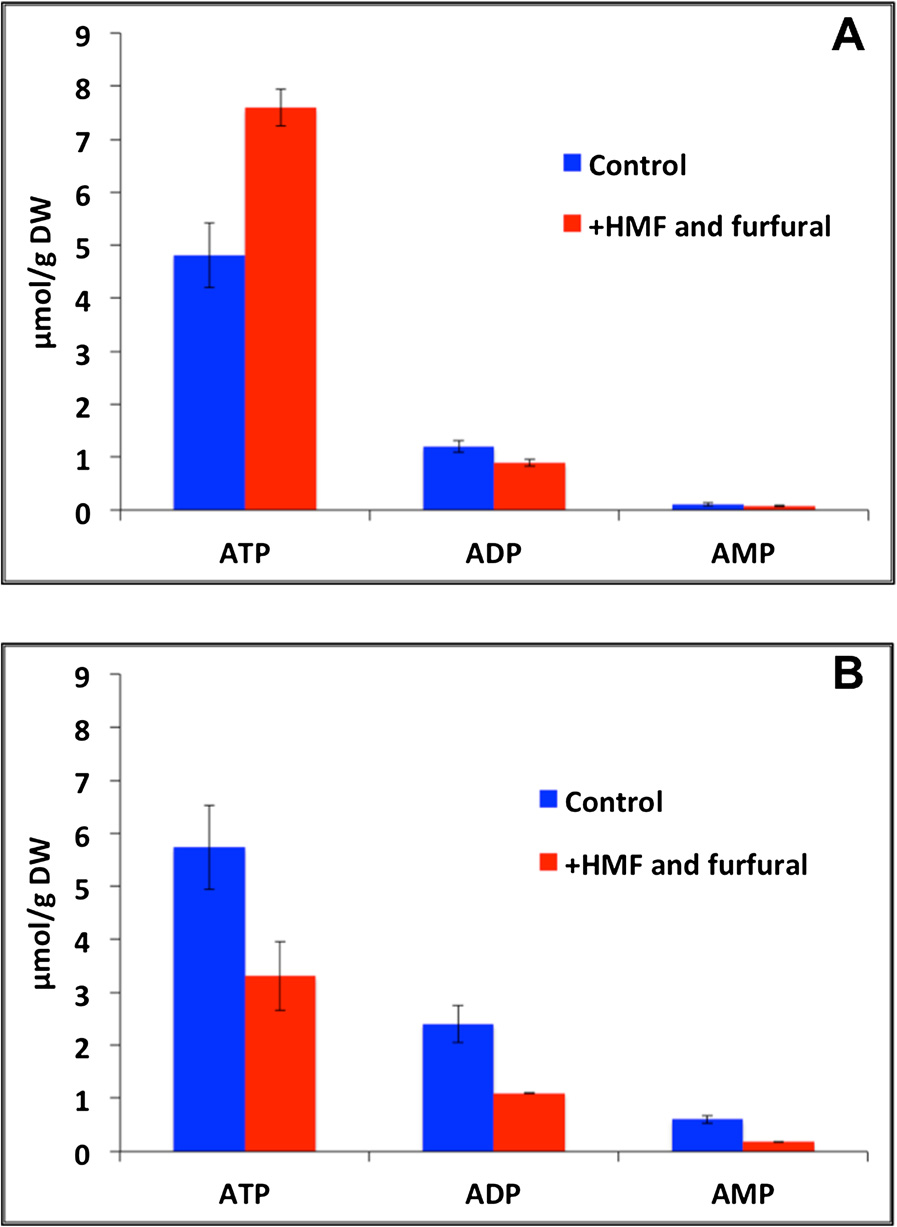
**Intracellular concentrations of the adenonucleotides ATP, ADP, and AMP.** Intracellular concentrations of the adenonucleotides ATP, ADP, and AMP 1 hour after pulsing of inhibitors in **(A)** the glucose consumption phase and **(B)** xylose consumption phase, respectively, compared with non-pulsed control cultivations. The columns represent data obtained from three independent cultivations and the error bars indicate the standard deviations*.* HMF: 5-hydroxymethylfurfural.

The energy state in terms of the intracellular concentrations of the adenonucleotides ATP, ADP, and AMP was quantified 1 hour after pulsing with inhibitors. Surprisingly, the intracellular concentration of ATP in the pulsed cultivation was significantly higher than in the non-pulsed control. The intracellular concentration of ATP was estimated at 7.60 μmol (g DW)^-1^ in the pulsed cultivations, whereas the concentration of ATP in cells in the non-pulsed cultivation was estimated at 4.81 μmol (g DW)^-1^ (Figure 4A). Also the intracellular concentration of ADP changed upon pulsing with HMF and furfural. The concentration of ADP was lower in cells in the pulsed cultivations (0.89 μmol (g DW)^-1^), compared with cells in the non-pulsed cultivations (1.20 μmol (g DW)^-1^) (Figure 4A). No significant difference was observed in the intracellular AMP concentrations between the two conditions. As a consequence of the altered ATP and ADP levels following the inhibitor-pulse, the energy charge was higher in cells in the inhibitor-pulsed cultivations (0.94) compared with the cells in the non-pulsed cultivations (0.88).

#### Transcriptome changes in response to HMF and furfural pulse in the glucose consumption phase

In order to study the transient changes at transcriptome level following pulsed addition of HMF and furfural, samples for RNA extraction were taken before and 1 hour after pulsing of inhibitors. By considering genes with a log_2_fold-change >1 and a false discovery rate (FDR) <0.01 as differentially expressed, 520 genes were found to be upregulated and 366 downregulated, respectively, 1 hour after pulsing of inhibitors in the glucose consumption phase. Functional analysis of the induced genes with Gene Ontology (GO) term finder revealed that 10% of the upregulated genes had oxidoreductase activity [Additional file 1: Table S1], a particularly interesting finding since the effect of HMF and furfural on redox metabolism was one of the targets in the present study. Both *ALD3* and *ALD4* encoding NAD(P)^+^-dependent acetaldehyde dehydrogenases were grouped under the oxidoreductase activity term. Conversion of acetaldehyde to acetate generates reducing power in the form of NAD(P)H, which could be used for detoxification of HMF and furfural. The upregulation of these genes could partly explain the increased acetate levels after pulsing. Several other genes coding for proteins with oxidoreductase activity potentially having the ability to convert HMF and/or furfural to less toxic compounds such as *AAD4*, *AAD6*, *AAD14*, *ARI1*, *BDH2*, *FDH1*, *OYE3*, *SFA1*, *GRE2*, *GRE3*, *YNL181W*, and *YKL071W* were also found to be upregulated [Additional file 1: Table S1].

Functional enrichment analysis using Munich Information Center for Protein Sequences (MIPS) functional categories indicated that 31 categories were significantly overrepresented among the induced genes [Additional file 1: Table S2]. Notably, genes related to C-compound and carbohydrate metabolism, metabolism of energy reserves, protein degradation, stress response, and detoxification were found among the enriched functional categories. As many genes were statistically significant (FDR <0.01) but did not change expression more than twofold, the reporter metabolites algorithm was applied to the upregulated gene set [23]. This method uses *P* values of differential expression and metabolic network topology as inputs to identify hot spots in metabolism around which transcriptional changes are apparent [23]. The analysis showed that metabolites such as glutathione, uridine diphosphate glucose (UDP-glucose), trehalose, trehalose-6-phosphate, and glucose-1-phosphate were among the top scoring reporter metabolites, again confirming that metabolism of energy reserves was induced after pulsing with HMF and furfural (Table 1). Glutathione, which functions in detoxification of ROS and redox buffering, was interestingly the highest scoring reporter metabolite.

**Table 1:** Top ten scoring reporter metabolites connected to transcriptome changes after inhibitor pulse in the glucose consumption phase

**Metabolite**	**Number of neighbors**	***P *** **value**
**Reporter metabolites (up)**		
Glutathione [c]	10	6.0E-05
UDP-glucose [c]	11	3.1E-04
D-galactose [e]	6	2.5E-03
Alpha,alpha-trehalose 6-phosphate [c]	4	3.7E-03
Alpha,alpha-trehalose [c]	4	6.0E-03
Alpha-D-glucose 6-phosphate [c]	8	8.2E-03
Oxidized glutathione [c]	4	6.6E-03
CDP-diacylglycerol [m]	4	8.6E-03
Alpha-D-glucose [c]	23	2.2E-02
D-glucose 1-phosphate [c]	6	1.2E-02
**Reporter metabolites (down)**		
AMP [c]	42	5.4E-09
ATP [c]	83	1.1E-08
5-phospho-alpha-D-ribose 1-diphosphate [c]	15	2.5E-06
L-glutamine [c]	17	6.6E-06
L-histidine [c]	7	8.2E-05
L-aspartate [c]	12	8.7E-05
CO_2 _[m]	10	1.3E-04
Isocitrate[m]	5	2.5E-04
Phosphate [c]	49	8.5E-05
Glycine [m]	4	9.6E-04

[c]: cytosolic; [m]: mitochondrial; [e]: extracellular; UDP-glucose: uridine diphosphate glucose.

Similar to the reporter metabolites algorithm, there is a corresponding method that investigates whether there are genes co-regulated by the same transcription factor [24]. Not surprisingly, reporter transcription factor analysis showed that the highest scoring transcription factors were all related to stress response. The highest scoring transcription factors encoded by *PDR1*, *PDR3*, *MSN2*, *MSN4*, *HSF1*, and *YAP1* are involved in multidrug resistance, environmental stress response, heat-shock response, and oxidative stress response, respectively [25-28].

Functional enrichment of the downregulated genes (log_2_fold-change >1, FDR <0.01) revealed 46 significantly enriched functional categories [Additional file 1: Table S3]. Several of these functional categories, and in particular amino acid metabolism, nucleotide metabolism, and protein synthesis, have previously been shown to include genes responsive to changes in specific growth rate [29]. Reporter metabolite analysis using the downregulated gene set as input identified significant changes around nodes such as AMP, ATP, 5-phospho-ribose-1-diphosphate (PRPP), and several amino acids (Table 1). Reporter transcription factor analysis highlighted *GCN4*, *BAS1*, and *PHO2* as significant for transcriptional control of the downregulated genes, which are involved in control of amino acid metabolism, purine metabolism, and phosphate metabolism, respectively [30,31].

### Pulse in xylose consumption phase

#### Changes in local yields of metabolic products

The strain used in the present study consumes glucose and xylose in a sequential manner, thus consuming the majority of the xylose when all glucose has been metabolized. To investigate the metabolic responses following an inhibitor pulse when the cells were utilizing xylose as the sole carbon source, HMF and furfural were added to cultivations approximately 4 hours after glucose had been exhausted (Figure 1B). The biomass concentration dropped during the first 2 hours after the pulse, after which it returned to levels similar as before the pulse. The specific furfural conversion rate was 12.7 times higher than the specific HMF conversion rate and both rates were 2.7-fold and 13.3-fold lower than the respective rates after pulsing in the glucose consumption phase. Similar to the analysis performed for the pulse in the glucose consumption phase, three phases were defined to explain the metabolic changes following the inhibitor pulse: xylose phase (period before the pulse, but after glucose had been consumed), co-conversion phase (period in which HMF and furfural were co-converted), and HMF phase (period when only HMF was converted). Progression from the xylose phase into the co-conversion phase resulted in significant increases in ethanol, carbon dioxide, glycerol, and acetate yields of 1.5, 1.3, 7.7, and 15.9-fold, respectively, whereas the xylitol yield decreased dramatically from 0.57 to 0.03 g g^-1^ (Figure 2B). In the HMF phase, the glycerol yield remained at a higher level than in the xylose phase while the other yields returned to similar levels as during the xylose phase, though the xylitol yield was slightly lower in the HMF phase than in the xylose phase (Figure 2B).

#### Redox and energy changes following inhibitor pulse

The redox cofactors were quantified 1 hour after pulsing inhibitors in the xylose consumption phase and compared to non-pulsed cultivations. The intracellular concentrations of the reduced redox cofactors NADH and NADPH decreased from 0.36 to 0.15 μmol (g DW)^-1^ and from 0.47 to 0.07 μmol (g DW)^-1^, respectively, 1 hour after pulsing of inhibitors, compared with the control cultivations (Figure 3B). The changes in the redox cofactor concentrations resulted in decreases of the catabolic and anabolic reduction charges from 0.11 to 0.04 and from 0.42 to 0.09, respectively.

Also the intracellular levels of adenonucleotides changed after pulsing with HMF and furfural. The concentration of ATP decreased from 5.74 to 3.31 μmol (g DW)^-1^, ADP decreased from 2.40 to 1.09 μmol (g DW)^-1^, and AMP decreased from 0.60 to 0.18 μmol (g DW)^-1^ compared to the control cultivations (Figure 4B). Even though the intracellular concentrations of the adenonucleotides decreased, the energy charge increased from 0.79 to 0.84 after the inhibitor pulse.

#### Transcriptome changes in response to HMF and furfural pulse in the xylose consumption phase

After applying the same thresholds as in the case for the inhibitor pulse in the glucose consumption phase, that is log_2_fold-change >1, FDR <0.01, 488 genes were found to be upregulated and 257 genes were downregulated 1 hour after pulsing in the xylose consumption phase compared to the non-pulsed cultivation. Similar to the case for the inhibitor pulse in the glucose consumption phase, GO term analysis identified oxidoreductase activity as a significantly enriched function among the induced genes. Overall, 8% of the upregulated genes coded for proteins with this function. Although these genes were coding for enzymes with oxidoreductase activity, their identities were different from the genes upregulated after pulsing in the glucose phase [Additional file 1: Table S4]. Interestingly, as many as seven genes involved in ergosterol biosynthesis were induced more than twofold, and five genes involved in sulfate assimilation were also upregulated. As these two processes are dependent on NADPH for reducing power either directly or indirectly, the upregulation of the genes involved in these processes may be a response for the cells to counteract the NADPH depletion resulting from HMF and furfural conversion. Another indication of NADPH deficiency was suggested from the 5.3-fold induction of *GDH1* coding for the NADPH-dependent glutamate dehydrogenase involved in reductive assimilation of NH_3_. Surprisingly, the major NADPH-generating enzymes in the pentose phosphate pathway (PPP) encoded by *ZWF1*, *GND1*, and *GND2* were not differentially expressed. On the other hand, *ALD6*, which encodes a cytoplasmic isoform of acetaldehyde dehydrogenase was upregulated 5.2-fold after pulsing of inhibitors in the xylose consumption phase, suggesting a higher demand for NADPH for reductive reactions. Induction of *ALD6* was also in line with the increased acetate yield following inhibitor addition. Moreover, it was found that *MAE1* was induced 3.9-fold. *MAE1* encodes the malic enzyme, which is a mitochondrial enzyme that converts malate to pyruvate with the concomitant production of NADPH [32]. Furthermore, *IDP2*, which encodes cytosolic isocitrate dehydrogenase, was upregulated 2.6-fold. Idp2p converts isocitrate to 2-oxoglutarate with the simultaneous production of NADPH.

Functional enrichment analysis using MIPS functional categories on the differentially expressed genes revealed a quite different response at transcriptome level compared to the pulse in the glucose phase. Interestingly, genes involved in amino acid metabolism, nucleotide metabolism, and rRNA processing were induced, that is, the opposite response compared to pulsing in the glucose phase [Additional file 1: Table S5]. Compared to the transcriptome response after pulsing inhibitors in the glucose consumption phase, relatively few genes related to stress response were induced after pulsing in the xylose consumption phase. Genes encoding ABC transporters involved in multidrug resistance and genes encoding proteins assigned to chemical agent resistance were upregulated. Relatively few functional classes were significantly enriched in the downregulated gene set [Additional file 1: Table S6]. Genes coding for proteins active in glycogen metabolism, fatty acid metabolism, and energy were found to be significantly downregulated. Reporter metabolite analysis using the induced genes as input showed that the sulfur-containing amino acids methionine and cysteine were among the highest scoring metabolites (Table 2). This was in accordance with the results from the functional enrichment analysis, which also indicated that sulfur metabolism was affected [Additional file 1: Table S5]. The analysis also showed that significant changes in gene expression occurred around metabolic pathways involving extracellular H^+^, ATP, AMP, and diphosphate. Reporter metabolites connected to the downregulated genes after pulsing in the xylose consumption phase were related to fatty acids, glycogen, and UDP-glucose (Table 2).

**Table 2:** Top ten scoring reporter metabolites connected to transcriptome changes after inhibitor pulse in the xylose consumption phase

**Metabolite**	**Number of neighbors**	***P *** **value**
**Reporter metabolites (up)**		
L-methionine[c]	14	1.2E-06
H(+)[e]	37	1.4E-06
ATP[c]	82	8.8E-07
AMP[c]	38	5.1E-06
Diphosphate[c]	50	6.3E-06
L-cysteine[c]	10	1.7E-04
L-methionine[e]	7	2.2E-04
L-glutamine[c]	16	1.1E-04
Coenzyme A[c]	23	1.5E-04
L-threonine[c]	8	5.0E-04
**Reporter metabolites (down)**		
Trans-2-C16-CoA[c]	4	5.4E-03
Trans-2-C14-CoA[c]	4	5.4E-03
Glycogen[c]	4	5.9E-03
Trans-2-C18-CoA[c]	3	9.5E-03
Trans-3-C16-CoA[c]	3	1.3E-02
Trans-3-C18-CoA[c]	3	1.3E-02
Trans-3-C14-CoA[c]	3	1.3E-02
UDP-glucose[c]	10	2.0E-02
CMP[m]	3	1.6E-02
FAD[m]	5	2.2E-02

[c]: cytosolic; [m]: mitochondrial; [e]: extracellular; CMP: cytidine monophosphate; FAD: flavin adenine dinucleotide; UDP-glucose: uridine diphosphate glucose.

Reporter transcription factor analysis indicated that *PDR1*, *YRR1*, *ECM22*, *STB5*, and *GCN4* were the highest scoring transcription factors controlling the upregulated genes. *PDR1*, *YRR1*, and *STB5* are all involved in regulation of genes encoding multidrug resistance proteins [33,34]. *ECM22* and *GCN4* are involved in sterol biosynthesis regulation and amino acid biosynthesis, respectively [30,35]. *CBK1*, *MOB2*, and *ACE2* were found to be reporter transcription factors of the genes downregulated after pulsing of HMF and furfural in the xylose consumption phase. They are all involved in controlling cellular polarity and morphogenesis [36].

#### Inhibitor pulses in glucose and xylose consumption phases elicited different transcriptome responses

**Figure 5:**
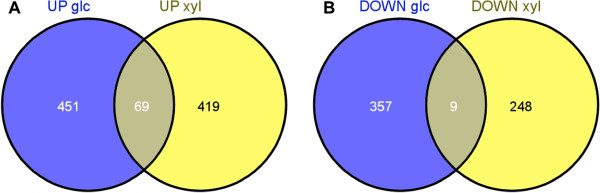
**Venn diagrams showing genes upregulated and downregulated. (A)** Genes upregulated and **(B)** downregulated after pulsing of HMF and furfural in the glucose and xylose consumption phase, respectively. Functional enrichment of the upregulated genes according to GO terms showed that they classified as molecular function unknown, transmembrane transporter activity, and oxidoreductase activity. GO term enrichment of the downregulated genes did not reveal any specific patterns*.* GO: Gene Ontology; HMF: 5-hydroxymethylfurfural.

In order to investigate the differences in the transcriptome responses following inhibitor pulses in the glucose and xylose consumption phases, the up- and downregulated genes fulfilling the criteria set previously (log_2_fold-change >1, FDR <0.01) were compared (Figure 5). Overall, 69 genes were found to be upregulated after pulsing in both the glucose and xylose consumption phases. Functional enrichment analysis indicated that genes coding for ABC transporters involved in multidrug resistance (*PDR5*, *YOR1*, *SNQ2*, and *PDR15*), detoxification, and unclassified proteins were significantly overrepresented among the upregulated genes. Moreover, a GO Slim Mapper functional analysis showed that the third most abundant functional group (after molecular function unknown and transmembrane transporter activity) was oxidoreductase activity, including the genes *ADI1*, *ARI1*, *CTT1*, *ERG27*, *GRE2*, *HBN1*, *MPD2*, and *PUT1*. Only nine genes were found to be downregulated in both experiments. Functional enrichment did not reveal any specific pattern among these genes.

## Discussion

In the present study, we investigated the physiological effects of HMF and furfural on a xylose-utilizing *S. cerevisiae* strain in terms of changes in metabolic product distribution, redox and energy state, and gene expression after pulsed addition of inhibitors in either the glucose or the xylose consumption phase. The physiological response differed in several aspects after inhibitor challenge in the two phases.

### Changes in local yields after inhibitor pulses

In general, the most pronounced changes in metabolism were observed in the co-conversion phase in which both HMF and furfural were converted simultaneously. Both HMF and furfural are reactive aldehydes known to have the potential to act as external electron acceptors in metabolism [37]. This property has been demonstrated to result in perturbations in the intracellular redox cofactor levels NAD(P)^+^ and NAD(P)H, which can be manifested in changes in yields of certain redox-related metabolic products [15,21,38]. Hence, it was not surprising that a reduction in the glycerol yield during co-conversion of HMF and furfural was observed, as these compounds act as external electron sinks, partly replacing glycerol as scavenger of surplus NADH produced in anabolic reactions. In addition, the biomass yield decreased, which would furthermore decrease the production of NADH in assimilatory reactions. Although this explanation seems coherent, the literature is somewhat ambiguous, as both increases and decreases in glycerol yield have been observed following addition of furfural and/or HMF to batch and chemostat cultivations, a fact that is likely the result of different cultivation methodologies and strain-specific differences [15,38-40]. On the other hand, when inhibitors were pulsed in the xylose consumption phase, the glycerol yield increased during the co-conversion phase. Increases in glycerol yield in this context have been hypothesized to result from non-specific stress responses [15]. Interestingly, the biomass concentration dropped drastically just after pulsing inhibitors in the xylose consumption phase. This can be indicative of utilization of energy reserves such as glycogen and trehalose, as it has been observed that *S. cerevisiae* can deplete these reservoirs rapidly after environmental perturbations [41]. Mobilization of glycogen and trehalose would thus provide reducing equivalents for conversion and energy in the form of ATP for repairing damage caused by HMF and furfural. In fact, genes involved in glycogen anabolism were downregulated and the carbon dioxide evolution rate increased immediately following the inhibitor pulse with no concomitant increase in xylose uptake, further supporting this hypothesis.

Pulses of inhibitors in both the glucose and the xylose consumption phase led to an increase in acetate yield. Acetate production from acetaldehyde generates reducing equivalents in the form of NAD(P)H, which could be used for furan aldehyde conversion [42]. However, it is not clear from the present data if the accumulation of acetate with the concomitant generation of NAD(P)H results in response to an increased NAD(P)H demand for HMF and furfural conversion, or if it results from acetaldehyde accumulation due to the competitive inhibition of alcohol dehydrogenases by the inhibitors. The latter hypothesis is likely, as alcohol dehydrogenases have been demonstrated to be capable of reducing furan aldehydes to furan alcohols [17].

In accordance with previous investigations [37], a dramatic drop in the xylitol yield was also observed in the present study after pulsing of HMF and furfural in the xylose consumption phase. Xylitol accumulation is a well-known problem in recombinant *S. cerevisiae* strains carrying the XR/XDH pathway, which is believed to result from the cofactor-bias of the involved enzymes [43]. It has previously been demonstrated that external electron acceptors such as acetoin and furfural can act as electron sinks by reoxidizing the NADH formed when xylitol is converted to xylulose [37].

#### Changes in redox and energy states after inhibitor pulses

Unexpectedly, the redox state in terms of the catabolic and anabolic reduction charges were unaffected after pulsing of HMF and furfural in the glucose consumption phase. As both HMF and furfural conversion require reducing equivalents in the form of NAD(P)H, the intracellular redox environment is expected to be more oxidized after inhibitor challenge, as would be indicated by a decrease in the catabolic and anabolic reduction charges. In fact, this was observed in a recent study in which HMF and furfural were added to the feed medium of a continuous cultivation [21]. The discrepancy of the results in the present study could be due to the transient nature of the response after pulsed addition of inhibitors, compared to the steady state in a continuous cultivation. Thus, it is possible that oscillations in redox cofactor levels take place on much smaller time-scales compared to the one investigated in the present study, where the sampling of intracellular metabolites was performed 1 hour after addition of HMF and furfural. Indeed, the capability of metabolism to respond rapidly to dynamic changes in the environment has been demonstrated by Theobald *et al*. [44], in which a pulse of glucose caused changes in the intracellular concentrations of the redox cofactors on a sub-minute scale.

Contrary to the results obtained after pulsing of inhibitors in the glucose consumption phase, both the catabolic and anabolic reduction charges were significantly decreased after pulsing of inhibitors during xylose consumption. This discrepancy could be an effect of the lower metabolic flux during xylose consumption, thus requiring a longer period of time to recover from the redox perturbation caused by HMF and furfural. As the redox cofactors are involved in numerous intracellular reactions, perturbations in the levels of these may have cell-wide effects, which was indicated in the transcriptome analysis where genes involved in redox cofactor-requiring processes such as ergosterol synthesis, sulfate assimilation, and ammonia assimilation were differentially expressed 1 hour after pulsing of inhibitors. The low xylose flux, and consequently, the lower production rate of reducing equivalents, may also explain the lower specific conversion rates of HMF and furfural in the xylose consumption phase compared with the specific conversion rates observed during the glucose consumption phase.

Interestingly, the energy charge was higher 1 hour after pulsing with inhibitors in the glucose consumption phase than in the control cultivation, which resulted from an increased intracellular concentration of ATP. The increased ATP concentration could have resulted from the reduction in biomass yield after addition of HMF and furfural, thus decoupling catabolism from anabolism, since glucose was still consumed, whereas only minor cell growth was observed. The reduced glycerol production could also explain the higher intracellular concentration of ATP, since glycerol synthesis is an ATP-consuming reaction. These results are in contrast to the ones we obtained in continuous cultivations [21], in which HMF and furfural stress resulted in decreased intracellular ATP levels. Again, the use of different cultivation methods (batch versus chemostat) is a realistic explanation for this discrepancy, and in particular the fact that the cells are growing at a constant specific growth rate in a chemostat.

In contrast to the results from the pulsing in the glucose consumption phase, the intracellular concentrations of all adenonucleotides were lower than the control 1 hour after pulsing in the xylose consumption phase. Despite this fact, the energy charge was slightly higher after pulsing inhibitors compared to the control. It is tempting to speculate that the reduction in the intracellular adenonucleotide pool resulted as a means of maintaining the energy charge at a high level despite reduced intracellular ATP levels caused by the presence of HMF and furfural. In fact, it has been demonstrated that pulsed addition of glucose, which is known to result in reduced intracellular ATP levels, is followed by the conversion of AXP into inositol by Amd1p and Isn1p in a two-step reaction, thereby stabilizing the energy charge [45-47]. It remains to be investigated whether similar mechanisms are active after pulsing of HMF and furfural in the xylose consumption phase. The difference in the responses in adenonucleotide levels between pulsing in the glucose or xylose consumption phases is likely related to the low metabolic flux during xylose conversion compared with when the cells are growing on glucose. As a consequence, energy-requiring repair mechanisms induced by furan aldehyde stress would have a bigger impact on the intracellular concentration of ATP, as the supply is lower in the xylose consumption phase. In fact, the low ATP generation rate during xylose conversion has been implicated in the lower acetic acid tolerance during xylose conversion, compared to when glucose is used as an energy source [13,48].

#### Transcriptome changes in response to HMF and furfural

Pulsing of inhibitors in the glucose consumption phase resulted in reduced specific growth rate and concomitantly, downregulation of transcripts known to be correlated with changes in specific growth rate, including genes involved in ribosome biogenesis, nucleotide metabolism, and amino acid synthesis [29]. On the other hand, genes involved in the environmental stress response (ESR) transcriptionally regulated by Msn2/Msn4 were upregulated in the response to HMF and furfural, but also these genes have been shown to be correlated with the specific growth rate. In fact, 89% of the genes upregulated as part of ESR following perturbations imposed by different stresses were also upregulated in response to reduced specific growth rate [29]. This confounding factor is problematic, as it is difficult to distinguish the cause and effect. In contrast to the transcriptome changes related to specific growth rate responsive genes observed after pulsing in the glucose consumption phase, pulsing of inhibitors in the xylose consumption phase resulted in an induction of genes involved in amino acid metabolism, nucleotide metabolism, and rRNA processing. This distinct response on transcriptional level is likely related to the fact that the cells are unable to grow in the xylose consumption phase.

A key finding of the inhibitor pulse experiment carried out in the xylose consumption phase was the concerted upregulation of several genes encoding proteins involved in sulfur assimilation, nitrogen assimilation, and amino acid biosynthesis. All these processes are known to require reducing equivalents in the form of NADPH, suggesting a link to the low intracellular NADPH concentration after pulsing of furan aldehydes during xylose consumption and gene expression. Since one way for the cell to increase the flux through a metabolic pathway is to synthesize more enzymes, we hypothesize that the cell compensates for the low intracellular NADPH levels by inducing genes involved in sulfur assimilation, nitrogen assimilation, and amino acid biosynthesis to maintain a flux in these processes. The influence of NADPH depletion on genes involved in sulfur assimilation has been demonstrated before by using the electron acceptor acetoin [49]. Limitations in sulfur assimilation have also been implicated in the response of *Escherichia coli* to furfural [50], but not in the case of *S. cerevisiae*.

Genes encoding proteins with oxidoreductase activity were found to be significantly enriched after pulsing of inhibitors in the glucose and xylose consumption phases, respectively. As it is generally thought that the reductive conversion of HMF and furfural are performed by oxidoreductases, this result was not surprising and is also in accordance with previous gene expression studies of HMF and furfural stress [39,51]. More surprising was the fact that the identities of the genes with oxidoreductase activity induced in the different phases were different. Of the 58 and 41 genes coding for proteins with oxidoreductase activity that were induced after pulsing of inhibitors in the glucose and xylose phases, respectively, only eight were upregulated in both cases. This pattern was also observed in general, as only 69 genes were upregulated in both cases (Figure 5).

Functional enrichment of the induced genes into MIPS functional categories showed that genes involved in pleiotropic drug resistance were upregulated under both conditions. Thus, it seems that the ability to transport toxic substances is indispensable for coping with HMF and furfural regardless of the carbon source. The high number of genes encoding unclassified proteins suggests that more activities are needed to successfully deal with the inhibitor challenge, a fact that calls for further studies characterizing the functions of these genes.

## Conclusions

In the present study, the effects of the lignocellulose-derived inhibitors HMF and furfural were characterized after pulsed addition in either the glucose or the xylose consumption phase in batch cultivations. By investigating the responses on metabolic products, redox and energy state, and gene expression, the following can be concluded about the distinct responses in the two phases:

When furan aldehydes are added in the glucose consumption phase, the cells can respond by decreasing the specific growth rate with the concomitant reduction of enzymatic activity in anabolic processes such as amino acid metabolism (NADPH- and energy-consuming), nucleotide biosynthesis (NADPH- and energy-consuming), and protein synthesis (ATP-consuming). The cessation of these processes occurs while no major effect on glucose uptake is apparent, consequently providing energy in the form of ATP and redox power in the form of NADPH that can be used for inhibitor conversion. Surplus energy generated is probably also stored in the form of trehalose and/or glycogen, as was indicated by the induced expression of genes involved in these processes after inhibitor pulse in the glucose consumption phase. However, during the xylose consumption phase, the cells are not growing (that is, they cannot stop growth to save energy and redox power) and are thus solely dependent on the low xylose flux for generation of energy and redox power. Hence, pulsing of inhibitors in the xylose consumption phase results in severe drainage of redox power, as was indicated from the reduction of catabolic and anabolic reduction charges, resulting in the upregulation of essential NADPH-consuming processes such as sulfate assimilation, nucleotide synthesis, amino acid synthesis, and ammonia assimilation. Judging from the results of the present study, improving the xylose utilization capability by metabolic engineering would potentially increase the inhibitor tolerance to HMF and furfural by increasing the supply of ATP and reducing power. In addition, by increasing the expression of ABC transporters and oxidoreductases identified as induced after pulsing of inhibitors in both the glucose and xylose consumption phases, more robust yeast strains for lignocellulosic bioethanol production could potentially be obtained.

## Methods

### Cell culture

#### Strain

The strain used in this study, VTT C-10883, was derived from VTT C-10880 (*MAT*α, *MAL2-8c*, *SUC2 ura3::XYL1 XYL2*, *XKS1::XKS1*)*.* VTT C-10880 was obtained in the genetic background of CEN.PK 113-1A (*MAT*α, *MAL2-8c*, *SUC2*) by overexpressing the endogenous xylulokinase encoding gene (*XKS1*) and integrating *S. stipitis* XR (*XYL1*) and XDH (*XYL2*) encoding genes in the *URA3* locus [52]. Prototrophy was restored by integrating an intact copy of *URA3* in 5′ of the heterologous construct and the resulting strain was named VTT C-10883.

#### Preparation of inoculum

Inoculum cultures were prepared from −80°C glycerol stocks by streaking on agar plates containing 10 g L^-1^ yeast extract, 20 g L^-1^ soy peptone, 20 g L^-1^ agar, and 20 g L^-1^ glucose. Colonies were transferred to 100 mL medium in a 500 mL Erlenmeyer flask. The composition of the medium was 20 g L^-1^ glucose, 20 g L^-1^ xylose, 5 g L^-1^ (NH_4_)_2_SO_4_, 3 g L^-1^ KH_2_PO_4_, 0.5 g L^-1^ MgSO_4_·7H_2_O, 1 mL L^-1^ vitamin solution, and 1 mL L^-1^ trace metal solution. The vitamin and trace metal solutions were prepared according to Verduyn *et al*. [53]. The cultures were grown for 24 hours at 30°C and pH 5 in a rotary shaker at 150 rpm.

#### Anaerobic batch cultivations

Precultured cells were inoculated at an OD_600_ of 0.2 in three to four individual bioreactors (Innova Biosciences, Cambridge, UK) using a final working volume of 1.5 L. The defined mineral medium was prepared according to Taherzadeh *et al*. [54] and contained 40 g L^-1^ glucose and 50 g L^-1^ xylose. The cultivations were carried out at 30°C and pH 5 with sparging of 0.1 VVM N_2_ to maintain anaerobic conditions. Ergosterol and Tween 80 were added at final concentrations of 0.01 g L^-1^ and 0.42 g L^-1^, respectively, to allow anaerobic growth. When approximately 40% of the glucose had been consumed, HMF (3.9 g L^-1^) and furfural (1.2 g L^-1^) were pulsed into the reactors (glucose phase). Samples for extracellular metabolites and cell dry weight (DW) were taken every 30 minutes until furfural had been consumed. Samples for intracellular redox cofactors and AXP were taken 1 hour after pulsing. Samples for transcriptome analysis were taken just before pulsing and 1 hour after pulsing. An experiment where no inhibitors were pulsed served as control. In a separate experiment, HMF (3.9 g L^-1^) and furfural (1.2 g L^-1^) were pulsed after 30% of the xylose had been consumed (xylose phase). The sampling was performed in the same way as for the cultivations pulsed in the glucose phase. An experiment in which no inhibitors were pulsed served as control.

### Analysis of extracellular metabolites

Extracellular metabolites were filtered through 0.2 μm syringe filters (VWR International, West Chester, PA, USA). The samples were stored at −20°C until analysis. Analysis of sugars and metabolites were performed using an HPLC system (Ultimate 3000, Dionex, Sunnyvale, CA, USA). Glucose, xylose, ethanol, xylitol, glycerol, acetic acid, HMF, and furfural were separated using an Aminex HPX87-H column (Bio-Rad Laboratories, München, Germany) with 5 mM H_2_SO_4_ as eluent. The column was operated at 60°C at a flow rate of 0.6 mL min^-1^. Ethanol, xylitol, glycerol, and acetic acid were detected with a refractive index detector Shodex RI-101 (Showa Denko, New York, NY, USA) while HMF and furfural were detected using an UV detector at 210 nm (Dionex).

### Determination of cell mass

The dry cell mass was determined by filtering 5 mL of culture broth through pre-dried 0.45 μm polyethersulfone membranes (Sartorius Stedim, Aubagne, France). The filters were washed with Milli-Q water (EMD Millipore, Billerica, MA, USA) and dried in a microwave oven at 120 W for 15 minutes. The filters were left to cool and dry further in a desiccator overnight and were subsequently weighed.

### Sampling of intracellular metabolites

#### Quenching

For sampling of intracellular metabolites, 5 mL samples were quenched according to Canelas *et al*. [55] in 25 mL pure methanol in pre-weighed tubes maintained at −40°C. The samples were allowed to cool for 3 minutes, after which the sample volumes were determined by weighing. The cells were then pelleted in a centrifuge (SIGMA Laborzentrifugen GmbH, Osterode, Germany) maintained at −20°C at 4,000 *g* for 5 minutes. Samples to be used for quantification of NAD(P)H were extracted instantly, whereas the other samples were flash-frozen in liquid nitrogen and stored at −80°C until analysis.

#### Extraction of NAD(P)H

The reduced redox factors are stable in alkaline conditions and were extracted as described in Moreira dos Santos *et al*. [56]. In short, 0.5 mL of 17% (v/v) alcoholic 1 M KOH was added to the samples, after which they were incubated at 70°C for 7 minutes in a water bath (Grant Instruments, Shepreth, UK).

#### Extraction of NAD(P)^+^

NAD^+^ and NADP^+^ were extracted by first suspending the cell pellets in 14% (w/w) HClO_4_ and thereafter disintegrating the cells with glass beads (diameter: 425 to 600 μm) in a bead mill. The acidic extracts were subsequently adjusted to pH 7 with 2 M KOH supplemented with 0.4 M imidazole.

#### Extraction of ATP, ADP, and AMP

ATP, ADP, and AMP were extracted by incubating the samples on ice for 15 minutes in the presence of 0.5 mL 0.52 M trichloroacetic acid (TCA) containing 17 mM EDTA, according to Lundin *et al*. [57]. The extracts were then centrifuged at 14,000 rpm for 3 minutes and subsequently neutralized with 2 M Tris-base.

### Analysis of intracellular metabolites

#### Quantification of NAD(P)^+^ and NAD(P)H

NADH and NADPH were first oxidized enzymatically since the reduced cofactors are unstable at non-alkaline conditions. The reaction was performed by adding 6 μL glutamate dehydrogenase (GLDH, 240 U mL^-1^) to extracts adjusted to pH 7 with substrate/buffer mixture containing (5 mM 2-oxoglutarate, 0.5 M HEPES, 0.5 M phosphate, and 30 mM NH_4_Cl). The reaction was allowed to take place at room temperature and was stopped with 3 M HClO_4_ after 20 minutes. The extracts were subsequently neutralized with 3 M KOH. The redox cofactors were quantified by enzymatic cycling according to Theobald *et al.*[44] and Vaseghi *et al.*[58] for NADH and NADPH, respectively, modified for use in 96-well plates. The assay mixture for determination of NAD^+^ contained 2 mM phenazine ethosulfate (PES), 0.5 mM thiazolyl blue tetrazolium bromide (MTT), 120 mM bicine, 1.61 M ethanol, and 50 μL sample in 300 μL total reaction volume. The assay mixture for determination of NADP^+^ contained 2 mM PES, 0.5 mM MTT, 120 mM bicine, 12.7 mM glucose-6-phosphate, 4.2 mM MgSO_4_·7H_2_O, and 50 μL sample. The reactions were initiated by addition of 5 μL alcohol dehydrogenase (ADH, 231 U mL^-1^) for determination of NAD^+^ or 5 μL glucose-6-phosphate dehydrogenase (G6PDH, 560 U mL^-1^) for determination of NADP^+^. The rate of formation of formazan, which correlates with the concentration of NAD(P)^+^, was measured at 570 nm in a plate reader (BMG Labtech GmbH, Ortenberg, Germany). Both reactions were performed at 30°C and standards of known concentrations were used for quantification.

#### Quantification of ATP, ADP, and AMP

Concentrations of adenonucleotides were determined via HPLC (Ultimate 3000, Dionex) equipped with a quaternary analytical pump (HPG-3400A, Dionex) fitted with a Luna® 5u C18(2) 100 Å LC column (150 x 4.6 mm, Phenomenex Inc., Torrance, CA, USA) kept at 20°C. The mobile phase consisted of acetonitrile (A) and tetrabutylammonium buffer (B) (0.005 M tetrabutylammonium hydrogen sulfate, 0.01 M Na_2_HPO_4_), pH 7.0. The pump was programmed to generate the following gradient: 6% A and 94% B (0 to 3 minutes), a linear increase of A to 25% and a linear decrease of B to 75% (3 to 16 minutes), 25% A and 75% B (16 to 22 minutes), a linear decrease of A to 6% and a linear increase of B to 94% (22 to 27 minutes), and 6% A and 94% B (27 to 35 minutes). The flow rate was 1 mL min^-1^. The detection was performed with a photodiode array detector (PDA-3000, Dionex) at 260 nm. Peak identities were confirmed by co-elution with standards and quantification was carried out by comparison using standard solutions of known concentrations.

### Transcriptome analysis

For transcriptome analysis, 5 mL samples withdrawn from three individual bioreactors for each condition were rapidly cooled on ice in pre-chilled Falcon tubes after which they were centrifuged at 4,000 *g* for 2 minutes. The cell pellets were frozen in liquid nitrogen and stored at −80°C until analysis.

The cells were mechanically disintegrated in a bead mill and total RNA was extracted and purified with RNeasy Mini Kit (Qiagen, Venlo, The Netherlands) according to the manufacturer’s instructions. The integrity of the RNA was assessed with an Agilent 2100 Bioanalyzer. Labeled RNA was produced using the GeneChip® 3′ IVT Express Kit (Affymetrix, Santa Clara, CA, USA) after which the labeled RNA was hybridized onto GeneChip® Yeast Genome 2.0 arrays. Three chips were hybridized for each condition. Staining and washing was performed in a GeneChip® Fluidics Station 450 (Affymetrix) and scanning of the chips were carried out in a GeneChip® Scanner 3000 7G (Affymetrix).

Data analysis was performed in R (version 2.15.3). The raw intensity data was background corrected with the robust multi-array average (RMA) algorithm [59], normalized with quantiles, and summarized using median polish. Differentially expressed ORFs were assessed using the limma package in R [60]. ORFs with a Benjamini-Hochberg FDR lower than 0.01 were considered as statistically significant and only ORFs with a log_2_fold-change greater than ±1 were taken into account. Gene set analysis was performed with the reporter metabolites algorithm [23] and by GO functional enrichment implemented in the R package piano [61]. Enriched functional categories were also identified with the MIPS functional catalogue (http://mips.helmholtz-muenchen.de/proj/funcatDB). Transcript data can be downloaded from the GEO database under the accession number [GSE:50182] (http://www.ncbi.nlm.nih.gov/geo/).

### Calculations

The yields were calculated based on the amount of consumed sugars (glucose and xylose) and statistical significance was determined with Student’s *t*-test in Microsoft Excel, 2011.

The energy charge was calculated from equation 1:

(1) $$E_{c}=\frac{\left[\mathrm{ATP}]+0.5\times [\mathrm{ADP}\right]}{\left[\mathrm{ATP}]+[\mathrm{ADP}]+[\mathrm{AMP}\right]}$$

The catabolic and anabolic reduction charges were calculated from equations 2 and 3, respectively:

(2) $$\frac{\left[\mathrm{NADH}\right]}{\left[\mathrm{NADH}]+[\mathrm{NA}D^{+}\right]}$$

(3) $$\frac{\left[\mathrm{NADPH}\right]}{\left[\mathrm{NADPH}]+[\mathrm{NAD}P^{+}\right]}$$

## Abbreviations

ABC: ATP binding cassette; ADH: Alcohol dehydrogenase; AXP: Collective term for ATP, ADP, and AMP; CMP: Cytidine monophosphate; DW: Dry weight; Ec: Energy charge; EDTA: Ethylenediaminetetraacetic acid; ESR: Environmental stress response; FAD: Flavin adenine dinucleotide; FDR: False discovery rate; G6PDH: Glucose-6-phosphate dehydrogenase; GLDH: Glutamate dehydrogenase; GO: Gene ontology; HMF: 5-hydroxymethylfurfural; HPLC: High performance liquid chromatography; MIPS: Munich information center for protein sequences; MTT: Thiazolyl blue tetrazolium bromide; NAD: Nicotinamide adenine dinucleotide; NADH: Nicotinamide adenine dinucleotide (reduced); NADP: Nicotinamide adenine dinucleotide phosphate; NADPH: Nicotinamide adenine dinucleotide phosphate (reduced); OD: Optical density; ORF: Open reading frame; PES: Phenazine ethosulfate; PPP: Pentose phosphate pathway; PRPP: 5-phospho-ribose-1-diphosphate; RMA: Robust multi-array average; ROS: Reactive oxygen species; TCA: Trichloroacetic acid; UDP-glucose: Uridine diphosphate glucose; VVM: Volume per volume per minute; XDH: Xylulose dehydrogenase; XI: Xylose isomerase; XR: Xylose reductase.

## Competing interests

The authors declare that they have no competing interests.

## Authors’ contributions

MA participated in the design of the study, conducted the experiments, analyzed the data, and wrote the manuscript. MB participated in the design of the study and commented on the manuscript. VRD participated in the wet laboratory experiments. LO participated in the design of the study and commented on the manuscript. All authors read and approved the final manuscript.

## Supplementary Material

Additional file 1: Table S1 Genes with oxidoreductase activity induced after pulsing of HMF and furfural in the glucose consumption phase. **Table S2.** Functional enrichment of genes upregulated in response to pulsing HMF and furfural in the glucose consumption phase. **Table S3.** Functional enrichment of genes downregulated in response to pulsing HMF and furfural in the glucose consumption phase. **Table S4.** Genes with oxidoreductase activity induced after pulsing of HMF and furfural in the xylose consumption phase. **Table S5.** Functional enrichment of genes upregulated in response to pulsing HMF and furfural in the xylose consumption phase. **Table S6.** Functional enrichment of genes downregulated in response to pulsing HMF and furfural in the xylose consumption phase. HMF: 5-hydroxymethylfurfural. Click here for file
